# Association of Hospital Matrons

**Published:** 1919-05-24

**Authors:** A. Lloyd Still, R. Cox Davies

**Affiliations:** President.; Hon. Secretary.


### THE EDITOR'S LETTER-BOX.

**[Correspondence on all subjects is invited, but we cannot in any way be responsible for the opinions expressed by our correspondents, who must give their name and address as a guarantee of good faith, but not necessarily for publication. Correspondents are reminded that brevity of style and conciseness of statement greatly facilitate early insertion.]**

### ASSOCIATION OF HOSPITAL MATRONS.

MISS COX DAVIES, the honorary secretary of the Association of Hospital Matrons, requests us to publish the following letter which has been sent to the Editor of the *British Journal of Nursing* :—

MADAM,—Our attention has been drawn to a paragraph appearing in this week's issue of the *British Journal of Nursing* headed : "Imitation is the Sincerest Form of Flattery." We write to inform you that the statement made therein—namely, that the constitution of the Matrons' Council of Great Britain and Ireland was used as a basis for that of the Association of Hospital Matrons—is incorrect, and it is to be regretted that this should not have been verified before publication. We now state, for your information, that though many members of this newly formed Association were doubtless at some period connected with the Matrons' Council of Great Britain and Ireland, the constitution of that Society was neither considered, mentioned, nor in the possession of any member of the committee dealing with this subject.

The inaccuracy of this statement is of little importance to us, and our only reason for requesting you to correct it is, lest by our silence, we appear ungrateful to the president of the Scottish Matrons' Association, whose kind assistance in drafting the constitution of the Association of Hospital Matrons we have already most gratefully acknowledged. We note in the concluding paragraph the recommendation that membership with the Matrons' Council of Great Britain and Ireland should have been sought, rather than the formation of another Association with kindred aims. We, on our part, deeply regret the need for this action, adding only, that had the Matrons' Council of Great Britain and Ireland fulfilled the ideals of its great founder there would have been no necessity to call into existence at this moment a truly representative body—able for that reason to take action on behalf of their profession at this crisis in its history.

A copy of this letter has been sent to the nursing press.
—We are, Madam, yours truly,

A. LLOYD STILL, *President.*

May 18, 1919. R. COX DAVIES, *Hon. Secretary.*

